# The Metal Chelators, Trientine and Citrate, Inhibit the Development of Cardiac Pathology in the Zucker Diabetic Rat

**DOI:** 10.1155/2009/696378

**Published:** 2009-04-15

**Authors:** John W. Baynes, David B. Murray

**Affiliations:** ^1^Department of Exercise Science, University of South Carolina, Columbia, SC 29208, USA; ^2^Department of Cell and Developmental Biology and Anatomy, University of South Carolina, Columbia, SC 29208, USA; ^3^Department of Pharmacology, University of Mississippi, Oxford, MS 38677, USA

## Abstract

*Purpose*. The objective of this study was to determine the efficacy of dietary supplementation with the metal chelators, trientine or citric acid, in preventing the development of cardiomyopathy in the Zucker diabetic rat. *Hypothesis*. We hypothesized that dietary chelators would attenuate metal-catalyzed oxidative stress and damage in tissues and protect against pathological changes in ventricular structure and function in type II diabetes. *Methods*. Animals (10 weeks old) included lean control (LC, *fa/+*), untreated Zucker diabetic fatty (ZDF, *fa/fa*), and ZDF rats treated with either trientine (triethylenetetramine) or citrate at 20 mg/d in drinking water, starting when rats were frankly diabetic. Cardiac functional assessment was determined using a Millar pressure/volume catheter placed in the left ventricle at 32 weeks of age. *Results*. End diastolic volume for the ZDF animals increased by 36% indicating LV dilatation (*P* < .05) and was accompanied by a 30% increase in the end diastolic pressure (*P* ≤ .05). Both trientine and citric acid prevented the increases in EDV and EDP (*P* < .05). Ejection fraction and myocardial relaxation were also significantly improved with chelator treatment. *Conclusion*. Dietary supplementation with trientine and citric acid significantly prevented structural and functional changes in the diabetic heart, supporting the merits of mild chelators for prevention of cardiovascular disease in diabetes.

## 1. Introduction

Cardiovascular disease is the primary cause of increased mortality in diabetes. Clinically, over 60% of diabetics succumb to heart failure. Moreover, the 5-year survival rate following the first documented heart injury is less than 50% [1]. These statistics underscore the importance of investigating more direct and effective treatment strategies for the ever increasing diabetic population. Recently, the transition metal chelator, trientine, was shown to improve cardiac extracellular matrix remodeling and cardiovascular function in streptozotocin-induced (type 1) diabetic rats [2]. In clinical studies, trientine administration for 6 months to a group of patients with type 2 diabetes also led to a decrease in left ventricular mass toward normal, concomitant with increased urinary copper excretion [3]. Similar protective effects on cardiovascular function are seen with breakers of advanced glycation end-products (AGE breakers) in both preclinical and clinical studies [4, 5]. However, these compounds and their degradation products are potent chelators, and there is evidence that chelation of transition metals is the primary mechanism of action of AGE breakers [6–9]. The beneficial effects of AGE inhibitors in treatment of diabetic complications are also attributed, in part, to their chelation activity and inhibition of oxidative stress [7, 8].

 Trientine and AGE breakers and inhibitors are potent chelators of copper, and to a lesser extent iron, and are thought to act by inhibition of metal-catalyzed oxidative stress. Oxidative stress is implicated in the development of the full range of diabetic complications, including nephropathy, neuropathy, retinopathy, and micro- and macrovascular disease [10–13]. Although both enzymatic and nonenzymatic reactions contribute to the increase in oxidative stress and inflammation in diabetes, nonenzymatic mechanisms are clearly important, not only directly by metal-catalyzed production of reactive oxygen species (ROS) [10, 11, 14], but also through glycoxidation and lipoxidation reactions [15, 16]. The transition metals, Cu and Fe, have also been linked to oxidative damage in other organ systems and diseases [13, 17–19], and in numerous studies, chelators have provided protection against development of diabetic complications in animal models of diabetes [17–20].

Accordingly, we designed this study to determine if the relatively inexpensive and well-tolerated chelators, trientine and citric acid, would alter the onset of cardiomyopathy in the Zucker diabetic rat, a model of type II diabetes, which accounts for more than 90% of the diabetic population. We show here that both of these mild chelators provide protection against the development of diabetes induced cardiomyopathy, supporting the merits of further research on chelation therapy for prevention and treatment of this and other diabetic complications. 

## 2. Materials and Methods

### 2.1. Experimental Design

All protocols were approved by the Institutional Animal Care and Use Committee of the University of South Carolina and conform to the *Guide for the Care and Use of Laboratory Animals*, published by the US National Institutes of Health (NIH Publication no. 85-23, revised 1996). All chemicals and reagents were purchased from Sigma-Aldrich (St. Louis, Mo, USA), unless otherwise indicated. All animals were housed two to a cage in a 12:12 hours dark/light cycle controlled room. Male Zucker diabetic fatty rats (ZDF *fa/fa*) and male Zucker lean control heterozygous litter mates (LC *fa/+*) were obtained at 6 weeks of age (~200 g) from Charles River Laboratories (Wilmington, Mass, USA). The ZDF is an inbred rat model of adult onset type II diabetes, characterized by gradual development of insulin resistance, hyperinsulinemia and hyperglycemia. All Animals were maintained on Purina 5008 rat chow with water ad libitum. At the age of 10 weeks, after confirming hyperglycemia by tail vein blood samples [378 ± 46 mg/dL; mean ± SD], the animals were divided into four groups: (1) lean control rats (LC; *n* = 7), (2) untreated ZDF rats (*n* = 10), (3) ZDF rats treated with trientine (ZDF + TN, *n* = 8) administered in drinking water (1 g/L) as previously reported [2], and (4) ZDF rats treated with citric acid (ZDF + CA, *n* = 4) administered at the same dose in drinking water (1 g/L). Left ventricular systolic and diastolic structural and functional parameters were assessed at 32 weeks of age, just prior to sacrifice. Plasma samples were collected, the heart and kidneys were removed, rinsed in ice cold PBS and weighed, and then portions either snap frozen in liquid nitrogen for biochemical analysis or placed in 4% paraformaldehyde fixative for histological analysis.

### 2.2. Left Ventricular Functional Assessment

**Figure 1:**
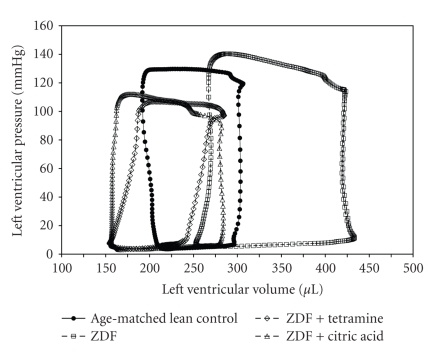
Representative left ventricle pressure/volume loops from lean control, untreated ZDF, ZDF + trientine, ZDF + Citric Acid animals. Beat by beat analysis of changes in LV hemodynamic parameters plotted along pressure (*y*-axis) and volume (*x*-axis) indicates a shift to the right in the ZDF animals suggesting LV dilatation, which is corrected by both trientine and citric acid.

As previously described, LV systolic and diastolic pressure and volume functional analysis were performed in anesthetized rats using a high-fidelity Millar pressure/volume (SPR-838; Millar Instruments, Houston, Tex USA) conductance catheter inserted into the LV via the right carotid artery [21]. Characteristic pressure-volume loops from these experiments are illustrated in Figure 1. For calibration, this measurement technique requires that a small amount (0.1 mL) of 15% saline be introduced into the blood volume of the anesthetized animal at the end of the experimental protocol as a correction factor for the blood-LV tissue interface. Data were analyzed using PVAN 3.5 Millar Instruments software.

### 2.3. Statistics

Statistical analyses were performed with Graphpad 5.0 software (Prism Inc., San Diego, Calif, USA). All grouped data are expressed as means ± SEM unless otherwise noted. Grouped data comparisons were made by one-way analysis of variance with intergroup comparisons analyzed using Bonferroni's posthoc testing. Non parametric Kruskal-Wallis test with Gaussian approximation was utilized to analyze the rank/scoring system of the hydronephrosis ratings. Statistical significance was taken to be *P* ≤ .05.

## 3. Results

### 3.1. Comparison of Body and LV Weights between Lean Control, Untreated ZDF, ZDF + Trientine, and ZDF + Citric Acid at 32 Weeks of Age

Compared to lean control animals, there was a significant reduction in whole body and LV weight in the ZDF and ZDF + CA groups at 32 weeks of age (Table 1). Body weight and LV weight were, on average, lower in the trientine treated group, but were not statistically different from LC. LV mass/BW ratios were similar (Table 1) in all groups. These trends were also evident when normalized to tibial lengths (data not shown). Mean arterial blood pressure was not appreciably different between lean control and ZDF (113 ± 5 versus 108 ± 6 mmHg; LC versus ZDF), or in the trientine or citric acid treated ZDF animals (119 ± 9 versus 94 ± 9 mmHg; ZDF + TN versus ZDF + CA, resp.). Lastly, blood glucose was increased to a comparable level in all the ZDF groups regardless of treatment (Table 1).

### 3.2. Left Ventricle Morphology and Function

**Figure 2:**
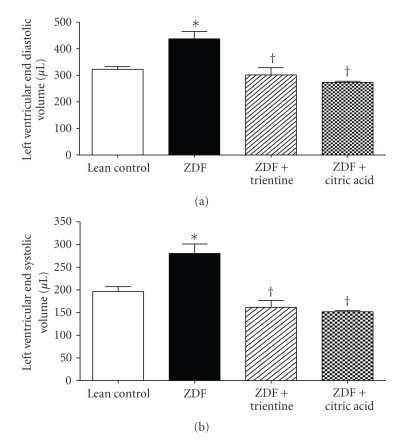
Comparison of (a) LV end diastolic volume and (b) end systolic volume between lean control and Zucker diabetic fatty (ZDF), ZDF + trientine, and ZDF + citric acid treated rats. Values are reported as mean ± SEM (*n* = 4–6 per group). ∗ denotes *P* ≤ .05 versus LC. † denotes *P* ≤ .05 versus ZDF.

Although heart rate was significantly reduced in the ZDF + CA treated group compared to LC, no differences in cardiac output were evident between any of the groups (Table 2). Stroke volume was significantly increased in the untreated ZDF group relative to LC (Table 2), but was not significantly affected by either chelator. End diastolic pressure (EDP) (Table 2) and end diastolic volume (EDV) (Figure 2(a)) were significantly increased in ZDF animals, compared to controls. In contrast, although end systolic pressure (ESP) was not increased in ZDF animals (Table 2), there was still a significant increase in end systolic volume ESV (Figure 2(b)). These increases in EDP, EDV, and ESV, which are common in type 2 diabetic patients, were completely prevented by treatment with either trientine or citric acid (Table 2 and Figure 2). 

**Figure 3:**
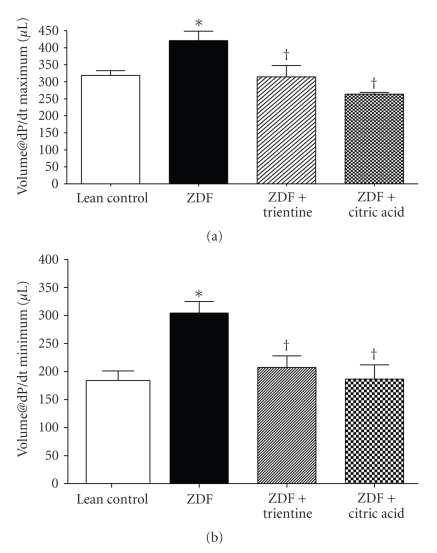
Evaluation of the LV chamber volumes at which (a) maximum and (b) minimum rate pressure product is generated between lean control and Zucker diabetic fatty (ZDF), ZDF + trientine, and ZDF + citric acid treated rats. Values are reported as mean ± SEM (*n* = 4–6 per group). ∗ denotes *P* ≤ .05 versus LC. † denotes *P* ≤ .05 versus ZDF.

Neither maximum nor minimum first derivative of LV pressure (dP/dt) (measurements of myocardial contractility and ventricular wall stiffness, resp.) was statistically different among groups (Table 2). However, compared to LC, the optimal volume for maximum dP/dt (Vol@dP/dt max) (i.e., the volume at which maximal contractility is generated) was significantly increased in the ZDF group (Figure 3(a)). This diabetes induced change in Vol@dP/dt max at 32 weeks of age was prevented in both the ZDF + TN and ZDF + CA groups (314 ± 33 versus 264 ± 5 *μ*L; resp. *P* ≤ .05 versus ZDF). Likewise the optimal volume for minimum dP/dt (Vol@dP/dt min) was also significantly increased in the untreated ZDF group compared to LC (Figure 3(b)). Changes in Vol@dP/dt min were also prevented by both trientine and citric acid (207 ± 20 and 186 ± 25 *μ*L, resp.; *P* ≤ .05 versus ZDF). Further, ejection fraction which was 34% in the untreated ZDF group was significantly improved, but not completely corrected by treatment with trientine (45%) or citric acid (43%). Lastly, in a effort to assess alterations in passive tissue property reflective of changes in the extracellular matrix, we analyzed the time constant of LV pressure decay (Tau) according to Weiss et al. [22]. Relative to age-matched LC, Tau, a measurement of the time needed for LV relaxation, was significantly increased in the ZDF group (12 ± 0.3 versus 15 ± 0.1 milliseconds, *P* ≤ .05). Tau values for ZDF + TN and ZDF + CA were not significantly different from LC, indicating attenuation in the overall stiffening or loss of compliance seen in the ventricle of the ZDF animals.

## 4. Discussion

Chelation therapy has a bad reputation in modern medicine. Indeed, the second citation in a Google search for “chelation therapy” yields the website “Quackwatch,” and an article titled: *Chelation Therapy: Unproven Claims and Unsound Theories*. However, chelation has proven effective in numerous studies in attenuating microvascular (renal, retinal, neuronal) as well as cardiovascular complications in animal models of diabetes [2, 3, 19, 23]. Adverse changes in vascular and ventricular structural and functional parameters are a major cause of cardiovascular failure in people with diabetes. Trientine, a chelator with a low toxicity profile, has been used therapeutically for many years for treatment of copper overload [13, 24]. Moreover, altered Cu (and iron) homeostasis is directly implicated in diabetic complications [20]. Alterations in iron homeostasis are also reported in diabetic subjects [25, 26], and treatment with desferrioxamine inhibits the development of neuropathy and microvascular disease in diabetic rats [27, 28]. Preclinical trials have also shown that short-term metal chelation by oral trientine can attenuate ventricular changes in diabetic patients [3, 29]. These previous studies demonstrated that trientine increased urinary copper excretion and also induced significant beneficial changes in left ventricular extracellular matrix structure.

In our studies of cardiac function, the data indicate that significant changes in LV chamber morphology occurred in the untreated ZDF animals as a diabetes-induced adaptation in an attempt to maintain overall function. The two chelators inhibited adverse changes in LV chamber morphology and relaxation. Specifically, type II diabetic rats treated with trientine or citric acid had significantly better in vivo left ventricular end systolic and end diastolic pressure/volume parameters than untreated animals, without a significant effect on blood glucose concentration. Additionally, both volumes at maximum and minimum dP/dt were significantly improved by trientine indicating that LV chamber underwent dilatation in order to maintain contractility. These results indicate that defective transition metal(s) homeostasis makes a significant contribution to pathogenic LV chamber remodeling in type II diabetes and that this cardiovascular pathology is prevented by chelation therapy. Although the experiments with citric acid were designed as exploratory and were limited in scope, they are reported here because, even in a small number of rats, citric acid had significant beneficial effects on both cardiac structure and function. Further studies on the effects of dietary citrate on iron and copper uptake and excretion in type II diabetes may provide some insight into the beneficial effects of citrus fruits in the human diet.

The difference in LV mass between the age-matched lean control and the untreated ZDF is an interesting observation. Indeed, changes in LV mass and or geometry greatly affect the overall function of the heart. Hypertrophy can be defined as either concentric (increase in wall thickness of the LV chamber typically characterized by an increase in the cross-sectional area of the myocytes due to parallel addition of sarcomeres) or eccentric (elongation of the LV chamber characterized by growth along the longitudinal axis of the myocytes due to in-series addition of sarcomeres). In either case, this is a progressive response to chronic physiologic stress such as alterations in cardiac output due to altered hemodynamic load on the heart [30]. In the current study, these changes were prevented by chelator treatment. Recent clinical studies on the correlation of left ventricular hypertrophy (LVH) and type II diabetes, based on echocardiography, indicate that there are a significant number of individuals who do not present with indication(s) of LVH [31–33]. Rather than viewing the changes in LV weight in the current study as a reduction in LV mass, we believe that the untreated ZDF animals are similar to the clinical condition, presenting with limited or inadequate hypertrophy at this time point.

Work by Cooper et al. suggests the beneficial effects of chelators may be at the extracellular level [2, 3]. In those studies trientine treatment was able to attenuate Cu-mediated activation of TGF-*β* and Smad, which subsequently suppressed collagen production, thereby limiting ECM accumulation common to the streptozotocin diabetic model. Further, trientine may not only act to sequester Cu for urinary excretion but also act to balance the activity or increase the expression of a number of oxidases (i.e., extracellular superoxide dismutase and semicarbazide sensitive amine oxidase) [2, 17]. Chelators also limit the formation of advance glycation endproducts (AGEs) [8, 17, 18, 34, 35]. AGE crosslinks directly affect ECM structure and function and also secondarily contribute to sustained activation of the receptor for AGEs (RAGE), leading to chronic NFkB mediated inflammatory responses, and ultimately to organ dysfunction [36–38]. Advancements in our understanding of metal-catalyzed oxidation in the diabetic setting and application of chelation therapy to inhibit this process may enhance our ability to limit the progression of cardiovascular disease in diabetes [6–8].

It should be noted that diabetic cardiomyopathy is often accompanied by the development of cardiovascular autonomic dysfunction. Although we were unable to assess that in this study, Schreihofer et al. [39] investigated the sympathetic baroreceptor reflexes in juvenile (7 weeks of age) and adult (13 weeks of age) lean control and obese (nondiabetic) Zucker rats. Their results indicated that in adult obese Zucker rats, acute changes in arterial pressure evoke smaller sympathetic responses compared to those observed in age matched lean counterparts. Further, baroreceptor reflex impairment included regulation of heart rate and sympathetic vasomotor control. However, a previous study by Schmidt et al. [40] analyzed the development of neuronal axonal dystrophy in the obese Zucker rat model 6 to 7 months after the onset of type II diabetes. In contrast to the streptozotocin induced model of hyperglycemia, the ZDF rat model of type 2 diabetes failed to develop the characteristic neuropathologic lesions of neuroaxonal dystrophy in the superior mesenteric sympathetic ganglia and ileal mesenteric nerves. Therefore, a limitation of this study may be the unappreciated effects of chelator therapy on diabetes induced changes to autonomic function. 

In summary, chelation of transition metals by trientine or citric acid significantly attenuated structural and functional changes in the heart in the Zucker type 2 diabetic rat. These observations support a previous work in the streptozotocin-induced type 1 diabetic rat and in clinical studies, suggesting that transition metal chelation can attenuate maladaptive cardiovascular remodeling leading to cardiac dysfunction in diabetes. Thus, a pharmacologic regimen incorporating Cu (and Fe) chelation is a potentially beneficial treatment strategy to prevent or attenuate the progression of cardiomyopathy in diabetes, even in the presence of chronic hyperglycemia. However, the question of whether this treatment improves remodeling or inhibits damage remains uncertain. 

## Figures and Tables

**Table 1:** Comparison of body weight, LV weight, and blood glucose in lean control, untreated ZDF, ZDF + trientine, and ZDF + citric acid at 32 weeks of age. All values are reported as average ± SD. ∗ denotes *P* ≤ .05 compared to lean control.

	Body weight (g)	LV weight (mg)	LV/body weight index	Blood glucose (mg/dL)
Lean control	456 ± 11	920 ± 32	2.2	105 ± 7
ZDF	358 ± 39*	796 ± 80*	2.0	392 ± 29*
ZDF + trientine	403 ± 50	825 ± 76	2.1	370 ± 54*
ZDF + citric acid	327 ± 17*	694 ± 28*	2.1	384 ± 51*

**Table 2:** Comparison of in vivo left ventricular functional parameters in lean control untreated ZDF, ZDF + trientine, and ZDF + citric acid at 32 weeks of age. Values are presented as mean ± SEM. *n* = 4–6 per group. ∗ denotes *P* < .05 versus age-matched lean control. † denotes *P* < .05 versus untreated ZDF.

	Heart rate (bpm)	Cardiac output (mL/min)	Stroke volume (*μ*L)	End diastolic pressure (mmHg)	End systolic pressure (mmHg)	Max dP/dt	Min dP/dt
Lean control	291 ± 9	38 ± 2	121 ± 5	7 ± 0.4	132 ± 7	7244 ± 332	−7533 ± 554
ZDF	279 ± 7	39 ± 3	150 ± 9*	10 ± 0.6*	135 ± 7	7423 ± 500	−6777 ± 518
ZDF + trientine	270 ± 12	35 ± 4	130 ± 9	7 ± 0.3^†^	136 ± 11	7184 ± 576	−7392 ± 376
ZDF + citric acid	248 ± 13*	31 ± 2	122 ± 5	7 ± 0.5^†^	129 ± 13	6509 ± 484	−6659 ± 707

## Abbreviations

AGE: Advanced glycation end-product
CA: Citric acid
EDV: End diastolic volume
EDP: End diastolic pressure
ESV: End systolic volume
LC: Lean control
LV: Left ventricle
ZDF: Zucker diabetic fatty (rat)
